# Rhizosphere Shifts: Reduced Fungal Diversity and Microbial Community Functionality Enhance Plant Adaptation in Continuous Cropping Systems

**DOI:** 10.3390/microorganisms12122420

**Published:** 2024-11-25

**Authors:** Jichao Li, Yingmei Zuo, Jinyu Zhang

**Affiliations:** Medicinal Plants Research Institute, Yunnan Academy of Agricultural Sciences, No. 2238 Beijing Road, Kunming 650221, China; ljc@yaas.org.cn

**Keywords:** rhizosphere microbiome, biochar, continuous cropping obstacles, microbial community functionality, *Fusarium*

## Abstract

Continuous cropping problems constitute threats to perennial plant health and survival. Soil conditioners have the potential to enhance plant disease resistance in continuous cropping systems. However, how microbes and metabolites of the rhizosphere respond to soil conditioner addition remains largely unknown, but this knowledge is paramount to providing innovative strategies to enhance plant adaptation in continuous cropping systems. Here, we found that a biochar conditioner significantly improved plant survival rates in a continuous cropping system. The biochar-induced rhizosphere significantly alters the fungal community, causing a decline in fungal diversity and the downregulation of soil microbial community functionality. Specifically, the biochar-induced rhizosphere causes a reduction in the relative abundance of pathogenic *Fusarium* sp. and phenolic acid concentration, whose variations are the primary causes of continuous cropping problems. Conversely, we observed an unexpected bacterial diversity increase in rhizospheric and non-rhizospheric soils. Our research further identified key microbial taxa in the biochar-induced rhizosphere, namely, *Monographella*, *Acremonium*, *Geosmithia*, and *Funneliformis*, which enhance soil nutrient availability, suppress *Fusarium* sp., mitigate soil acidification, and reduce phenolic acid concentrations. Collectively, we highlight the critical role of regular microbial communities and metabolites in determining plant health during continuous cropping and propose a synthetic microbial community framework for further optimizing the ecological functions of the rhizosphere.

## 1. Introduction

Continuous cropping obstacles (CCOs) have become a staple in the pursuit of agricultural intensification. However, they lead to imbalances in rhizosphere ecology and heightened plant vulnerability to diseases, especially those caused by soil-borne pathogenic *Fusarium* [1]. Root-harvest-targeted medicinal plants (approximately 70%), e.g., *Panax notoginseng* [2], *Panax ginseng*, *Angelica sinensis*, and *Rehmannia glutinosa* [3], experience significant challenges from continuous cropping obstacles (CCOs) that lead to crop failure. CCOs constitute major threats to the yield and properties of medicinal plants and sustainable agriculture. Soil conditioners have shown the potential to improve plant disease resistance in continuous cropping systems. However, it is still unknown how a soil conditioner affects microbial communities and metabolites, which are crucial for plant health [4].

The rhizosphere, a hub of root activities, microorganisms, and metabolic exchanges, is crucial for driving plant growth, resistance, and evolution. It has been described as the second genome of plants and the first line of defense against pathogens [5]. Plants manipulate the soil microbiota by secreting bioactive molecules into the rhizosphere that attract plant-growth-promoting rhizobacteria (PGPR) and perform essential functions, such as nitrogen fixation, nutrient mobilization, and disease suppression, thereby enhancing soil microbial productivity and nutrient availability [6]. However, this process can also inadvertently promote soil-borne diseases, leading to continuous cropping obstacles (CCOs) that threaten plant health and agricultural economics.

In the rhizosphere, metabolites that favor pathogen preferences can enhance their survival and colonization. For instance, cinnamic acid has been shown to increase fusaric acid secretion by *F. oxysporum*, increasing plant susceptibility to infection [7]. Additionally, syringic acid can induce a shift in the tobacco rhizosphere microbial community from beneficial to detrimental, contributing to CCOs [8]. Organic acids can also enhance toxin production and H_2_O_2_ secretion by pathogens, weakening the antagonistic capabilities of PGPR and promoting pathogenic fungal growth [9]. Pathogens can manipulate host plants to release specific root exudates that amplify their virulence and produce chemo-sensory autotoxic substances. For example, *F. oxysporum* infection alters the expression of genes related to phenolic acid synthesis in host plants, leading to the increased synthesis and accumulation of phenolics in the rhizosphere [10].

Thus, the presence of pathogens and their “preference-type” rhizosphere metabolites in continuous cropping systems potentially accelerates the threat to rhizosphere microbial ecology. Rhizosphere microecological imbalances can significantly impede plant immunity and survival, presenting a substantial challenge in agricultural practices. In this study, we investigate the effects of soil conditioners on fungal and bacterial dynamics in both rhizosphere and bulk soils and their implications for soil health and plant survival. We further identify biomarkers and analyze their relationships with microbial communities, soil nutrients, and autotoxicity, understanding the critical role of rhizosphere microbes and metabolites in enhancing plant adaptation in continuous cropping systems.

## 2. Materials and Methods

### 2.1. Experimental Materials

Test soil: The potted experiment commenced in a greenhouse in Kunming County, Yunnan Province, China. The average humidity in the greenhouse ranged from 68 to 75%, with an average temperature of 17 to 21 °C. The continuous cropping soils for the experiment were collected following the harvest of *Panax notoginseng* (Burk.) F.H. Chen after 3-yr continuous cultivation in Wenshan County, Yunnan Province, China. The continuous cropping soil in this region is classified as red soil. Its initial physical and chemical properties included a pH of 5.7 and total N, total P, total K, and organic matter levels of 0.53, 0.61, 5.80, and 12.436 g/kg, respectively. Source of seedlings: We utilized *P. notoginseng* as a model plant, which causes more CCOs than other crops, thereby having a destructive impact on the survival rates of seedlings and leading to the complete loss of seedlings. The *P. notoginseng* seeds were chosen based on vigor, structural integrity, and the absence of insect infestation. The seeds, along with the seedling substrate, underwent a sterilization process. Subsequently, the seeds were sown and cultivated into seedlings, under uniform management, over a period of one year.

### 2.2. Strategy Design: Growth, Disease Development, and Soil Collection

We designed three strategies, i.e., biochar, grass ash, and corn stover. The biochar selected was rice husk charcoal with a total nano-pore size of 2.1 cm × 3 $g^{-1}$. Grass ash was derived from the combustion residues of rice straw and had a total ash content exceeding 80 percent. The chosen corn stover was the leaves of the corn plant, which had undergone natural maturation and desiccation. The experimental substrates, biochar, grass ash, and maize stover, were subjected to a sieving process using a sieve with a mesh size of 20.

**Figure 1:**
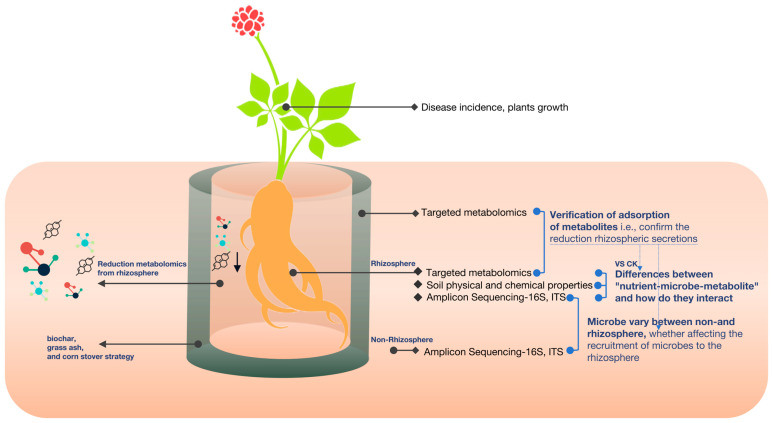
Materials and methods in experimental design.

We used the root bag method for the experiment. Small and large bags were sewn with 25 µm mesh diameter nylon mesh. The small bags were filled with *P. notoginseng* seedlings planted in the test soil. The three strategic substances were added in amounts of 50 g to a large bag (14 × 10 cm) and a small bag (12 × 8 cm), and 200 g of test soil with seedlings was placed into the large bag, forming an interlayer with three adsorption effects. Then, the large bag was placed in a pot (diameter 28 cm, height 40 cm), and the outside was filled with the same test soil (without the added substances). A control was set up where the interlayer was the same test soil (Figure 1). The potting unit was configured to include 30 pots per treatment group. After a particular growth stage, we investigated the plants’ growth and seedling survival rates [11]. Ten months after planting, ten soil cores with a depth of 10 cm were randomly selected from each pot, and these ten soil cores were thoroughly mixed into a single composite soil sample as non-rhizosphere soil. Excess soil on the roots was discarded by gently shaking the plants, and the remaining soil particles attached to the root surface were collected as rhizosphere soil. Samples of rhizosphere soil, non-rhizosphere soil, and interlayer material were collected and stored at −80 °C to facilitate further experimental analyses.

### 2.3. Profiling Non-Rhizosphere and Rhizosphere Fungi and Bacteria

Sequencing was performed using Illumina MiSeq, a second-generation high-throughput sequencing platform. The concentration of DNA was verified with a NanoDrop spectrophotometer and agarose gel electrophoresis. The genomic DNA was used as a template for PCR amplification with barcoded primers and Tks Gflex DNA Polymerase (Takara). For bacterial diversity analysis, V3–V4 (or V4–V5) variable regions of 16S rRNA genes were amplified with the universal primers 343 F and 798 R (or 515F and 907R for the V4–V5 region). For fungal diversity analysis, the ITS I variable region was amplified with the universal primers ITS1F and ITS2. The raw sequencing data were in FASTQ format. Paired-end reads were then preprocessed using Trimmomatic software (0.39) to detect and cut off ambiguous bases (N). Low-quality sequences with average quality scores of less than 20 were also removed using the sliding window trimming approach. After trimming, paired-end reads were assembled using FLASH software (2.2.15). The parameters of the assembly were as follows: 10 bp of minimal overlap, 200 bp of maximum overlap, and a maximum mismatch rate of 20 percent. The sequences were further denoised as follows: Reads with ambiguous or homologous sequences or less than 200 bp were discarded. Reads with 75 percent of bases above Q20 were retained. Then, reads with chimeras were detected and removed. These two steps were achieved using QIIME software (version 1.8.0). Clean reads were subjected to primer sequence removal and clustering to generate operational taxonomic units (OTUs) using Vsearch software (2.29.1) with a 97 percent similarity cutoff. The representative read of each OTU was selected using the QIIME package. All representative reads were annotated and blasted against the Silva database Version 138 (16 s/18 s rDNA) using the RDP classifier (the confidence threshold was 70 percent). All representative reads were annotated and blasted against the Unite database (ITS rDNA) using BLAST (2.15.0) [12].

### 2.4. Quantitative Wide-Target Metabolome Detection of the Rhizosphere Soil and Interlayer Metabolites

Sample preparation and extraction. After thawing the samples from the refrigerator at −80 °C, they were mixed by vortexing for 10 s. After mixing, 2 mL of each sample was added to a centrifuge tube, after which the sample was immersed in liquid nitrogen. The sample was placed into the lyophilizer for freeze-drying after it was completely frozen. After the samples were completely lyophilized, 200 µL of a 70 percent methanol internal standard extract was added. After centrifugation (12,000 r/min, 4 °C) for 3 min, the mixture was swirled for 15 min. The supernatant was filtered through a microporous filter membrane (0.22 µm) and stored in a sample flask for LC–MS/MS analysis.

UPLC conditions. The sample extracts were analyzed using a UPLC-ESI-MS/MS system (UPLC, SHIMADZU Nexera X2 (Shimadzu Corporation, Kyoto, Japan); MS, Applied Biosystems 4500 Q TRAP (AB SCIEX, Framingham, MA, USA)). The analytical conditions were as follows: UPLC: column, Agilent SB-C18 (1.8 µm, 2.1 mm × 100 mm). The mobile phase consisted of solvent A, pure water with 0.1 percent formic acid; solvent B, acetonitrile with 0.1 percent formic acid. Sample measurements were performed with a gradient program that employed starting conditions of 95 percent A, 5 percent B. Within 9 min, a linear gradient to 5 percent A, 95 percent B was programmed, and a composition of 5 percent A, 95 percent B was maintained for 1 min. Subsequently, the composition was adjusted to 95 percent A, 5.0 percent B within 1.1 min, which was maintained for 2.9 min. The flow velocity was set to 0.35 mL/min; the column oven was set to 40 °C; and the injection volume was 4 µL. The effluent was alternatively connected to an ESI-triple quadrupole-linear ion trap (QTRAP)-MS [13].

ESI-Q TRAP-MS/MS. The ESI source operating parameters were as follows: source temperature, 550 °C; ion spray voltage (IS), 5500 V (positive ion mode)/−4500 V (negative ion mode); ion source gas I (GSI), gas II (GSII), and curtain gas (CUR), 50, 60, and 25 psi, respectively; and high collision-activated dissociation (CAD) pressure. Instrument tuning and mass calibration were performed with 10 and 100 µmol/L polypropylene glycol solutions in the QQQ and LIT modes, respectively. QQQ scans were acquired as MRM experiments with the collision gas (nitrogen) set to medium. DP (declustering potential) and CE (collision energy) for individual MRM transitions were determined with further DP and CE optimization. A specific set of MRM transitions was monitored for each period according to the metabolites eluted within this period.

### 2.5. Effects on Rhizosphere Microbial Community Function

For PICRUSt analysis, we followed the suggested methods for OTU selection with Greengenes 13-5 using Galaxy One UI 6.1.1. PICRUSt (Phylogenetic Investigation of Communities by Reconstruction of Unobserved States) is a bioinformatics tool designed to predict the functional abundances of microbial communities based on marker gene sequences. It infers the metabolic potential of bacteria and archaea. The workflow of PICRUSt2 includes the following steps: The study sequences (OTUs and ASVs) are placed onto a reference tree using tools like HMMER, EPA-ng, and GAPPA. Core hidden state prediction functions are implemented using the R package castor. Metagenome profiles are generated and stratified based on contributing sequences [14]. The predicted gene family abundances were analyzed using the Kyoto Encyclopedia of Genes and Genomes ethology group count level 3, and Story FDR in STAMP software 2.1.3 was used to avoid Type-I errors. KEGG function prediction, enzyme classification number EC, and COG protein prediction were performed; differences were determined according to the Kruskal–Wallis algorithm, and the different results were selected to construct a heatmap map. Linear differential analysis effect size (LEfSe) was used to predict up- and downregulated functions and construct a differential heatmap [15].

### 2.6. Soil Physicochemical Analysis

The content of organic matter was determined by the potassium dichromate volumetric method. After acid dissolution, total nitrogen was determined by a SEAL-AA3 continuous flow analyzer, total phosphorus was determined by molybdenum–antimony colorimetry, and total potassium and available potassium were determined by a flame photometer. The available nitrogen content was determined via the alkali-hydrolyzed diffusion method, and the available phosphorus content was determined via the sodium bicarbonate method. The cation exchange capacity (CEC) was determined by the neutral ammonium acetate method [16].

### 2.7. Investigating Potential Interactions Among Biomarkers, Soil Nutrients, Metabolites, and Pathogens

Linear differential analysis effect size (LEfSe) was used to identify the up- and downregulated microorganisms and to construct a heatmap of the differentially abundant genera. To perform correlation and model prediction analyses, correlations between biomarker microorganisms and other microorganisms, soil nutrients, phenolic acids, and pathogens were evaluated. RDA and mapping were used. Based on two (or three) histological quantitative files, a correlation chord diagram, a cluster heatmap, and a network interaction diagram were drawn [17].

### 2.8. Statistical Analysis

After data processing, a community composition histogram was drawn to show the community structure distribution. The species richness and distribution uniformity of the samples were evaluated, the alpha diversity was analyzed, and a boxplot plot was drawn. The significance of the differences in the diversity indices among the different groups was calculated through plot analysis (Wilcoxon algorithm). Microbial multivariate statistical analysis: The difference in species (OTU or phylum, genus, and species level) between different groups was calculated through a statistical algorithm (Wilcoxon), the differentially upregulated and downregulated microorganisms were identified through linear differential analysis effect size (LEfSe), and a differential species heatmap was drawn. After the data were processed, a histogram was drawn to show the distribution of the community structure. The species richness and distribution uniformity in the samples were evaluated, and the alpha diversity was analyzed, which was then represented using a boxplot. The significance of the diversity index was calculated for the different groups through plot analysis (using the Wilcoxon algorithm) [18].

## 3. Results

### 3.1. Fungal Communities Are More Highly Affected by Rhizosphere Shifts than Bacteria

To explore the impact of soil conditioners on microbial communities and identify strategies for optimizing microbial structure, we utilized three proven approaches: biochar amendment, grass ash application, and corn stover incorporation. Our experimental design contrasts non-rhizosphere and rhizosphere soils.

**Figure 2:**
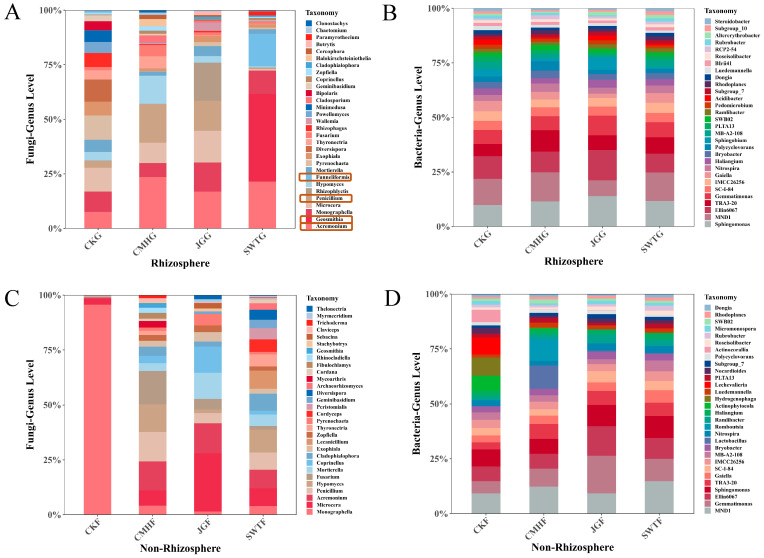
Comparative community structure of fungi and bacteria at the genus level. (**A**,**B**) Fungal and bacterial community structure in the rhizosphere with SWTG (biochar), CMHG (grass ash), JGG (corn stover), and CKG (control). The orange boxes indicate the genera with significant alterations. (**C**,**D**) Fungal and bacterial community structures in the non-rhizosphere. Similarly, SWTF (biochar), CMHF (grass ash), JGF (corn stover), and CKF (control) are shown.

Plant growth and seedling survival rates differed among the strategies, with the biochar strategy having the most favorable phenotype and seedling survival (*p* < 0.01) (Figure S1). Similarly, we found that the microbial community composition displayed high variability between fungal communities and even between the non-rhizosphere and rhizosphere soils, highlighting the microbiome heterogeneity of soil when the rhizosphere shifts (Figure 2). The bacterial and fungal communities exhibited significant differences; the vast majority of the bacterial communities exhibited structural similarities in response to the different strategies (Figure 2B,D), and the fungal communities were more strongly affected than bacterial communities (Figure 2). In the non-rhizosphere, all the strategies had greater fungal richness than the control group, indicating that rhizosphere shift limits fungal microbial recruitment to the rhizosphere (Figure 2C).

The *Acremonium* genus was highly represented across all three systems, while the richness of the *Penicillium* genus was greatest in the CMHG and corn stover strategies (JGG). Notably, the biochar strategy (SWTG) revealed a distinct trend of specific genera aggregating in the rhizosphere, including *Geosmithia* spp. and *Funneliformis* spp. (Figure 2A).

### 3.2. Fungal Diversity Reverses Under Soil Conditioner Addition, Decreasing in the Rhizosphere and Increasing in the Non-Rhizosphere

**Figure 3:**
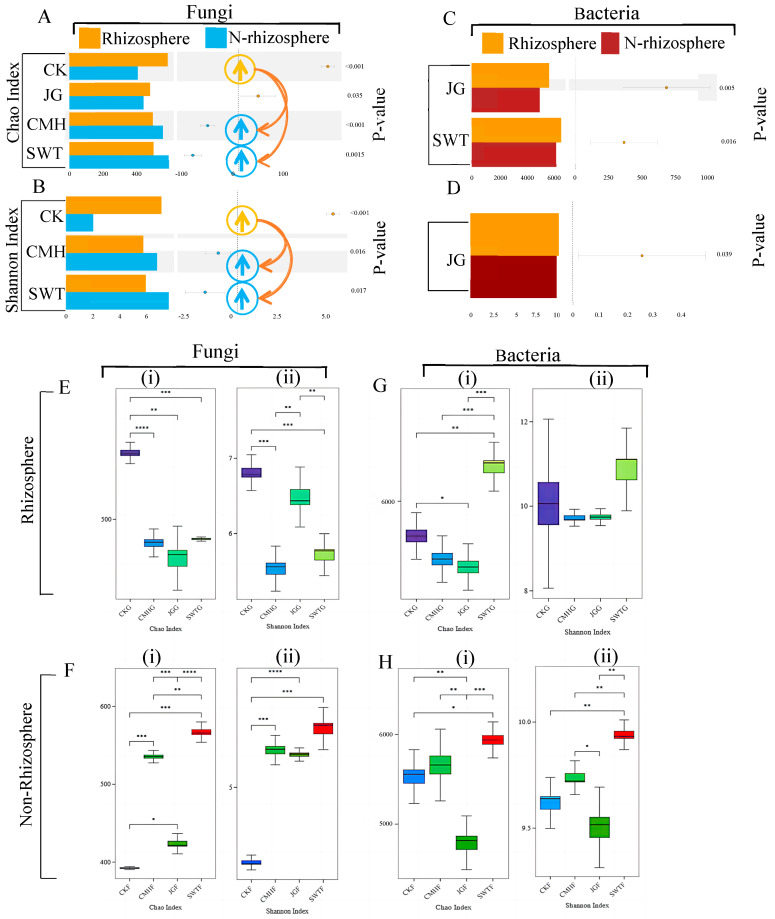
Comparison of rhizosphere and non-rhizosphere fungal and bacterial diversity at the genus level. (**A**,**B**) A fungal Chao and Shannon index paired map between the rhizosphere and non-rhizosphere. It only shows significant differences between strategy pairs. Higher diversity in the rhizosphere than in the non-rhizosphere is indicated by yellow arrows. Higher diversity in the non-rhizosphere than the rhizosphere is indicated by blue arrows. SWT (biochar), CMH (grass ash), JG (corn stover), and CK (control). (**C**,**D**) Bacterial Chao and Shannon index paired map between the rhizosphere and non-rhizosphere. It only shows significant differences between strategy pairs. (**E**,**F**) Fungal Chao (**i**) and Shannon (**ii**) indices with soil conditioners in the rhizosphere and non-rhizosphere. (**G**,**H**) Bacterial Chao (**i**) and Shannon (**ii**) indices with soil conditioners in the rhizosphere and non-rhizosphere. *, *p* < 0.05; **, *p* < 0.01; ***, *p* < 0.001; ****, *p* < 0.0001.

Next, we performed a paired test comparing non-rhizosphere and rhizosphere microbial diversity to examine how microbial communities near or far from the rhizosphere vary. Our findings highlight significant differences in microbial diversity with soil conditioner addition, particularly for fungi, indicating a reversal from the control group. In the control, the rhizosphere exhibited higher fungal diversity than the non-rhizosphere, whereas with soil conditioner addition, the fungal diversity of the rhizosphere was lower than that of the non-rhizosphere, as evidenced by the biochar and grass ash treatments (*p* < 0.001) (Figure 3A,B). Bacterial diversity showed no significant differences between the non-rhizosphere and rhizosphere soils in the control group (Figure 3C,D), but notable variation was observed in the biochar treatment group (Figure 3C).

We performed a detailed analysis of the differences in microbial diversity among all the strategies. Fungal diversity followed a similar trend across all strategies for reducing rhizosphere metabolites (Figure 3E,F), while bacterial diversity varied. We observed greater diversity with the biochar strategy (SWTG) in both the rhizosphere and non-rhizosphere and lower diversity with the corn stover strategy (JGG), which was significantly different from the control (*p* < 0.05) and biochar strategy (*p* < 0.001), and almost no change occurred with the grass ash strategy (CMHG) (Figure 3G(i),H(i)).

Overall, the biochar strategy decreased the fungal diversity and increased the bacterial diversity of the rhizosphere, improving the microbiological environment.

### 3.3. Rhizosphere Shifts Downregulated Metabolic Pathways, Enzyme Activity, and Protein Expression

**Figure 4:**
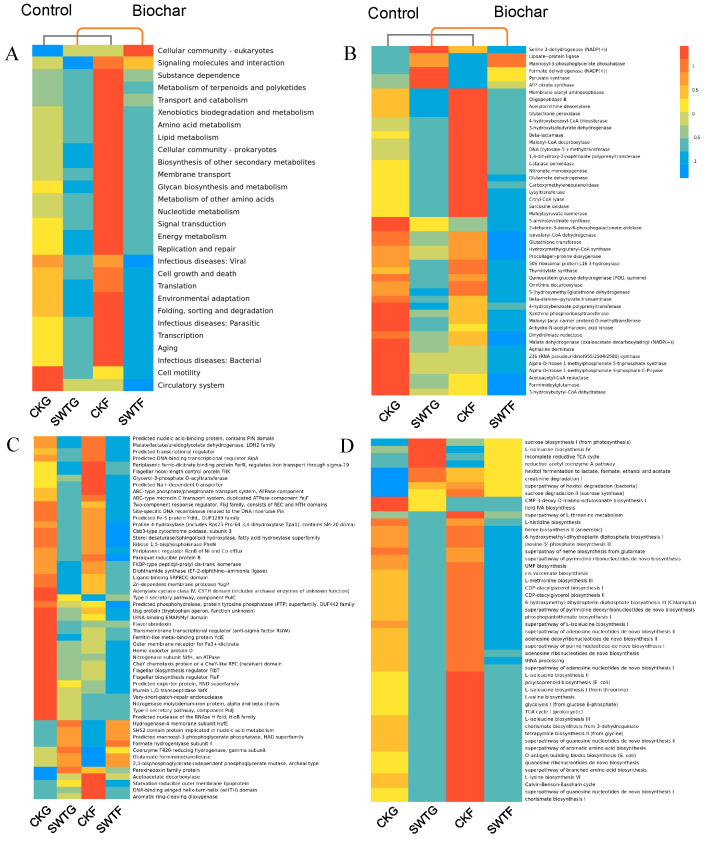
Biochar-induced soil microbial functional abundance variations in the rhizosphere and non-rhizosphere. (**A**) Hierarchical cluster heatmap of KEGG level 2 functional predictions. (**B**) Enzyme activity difference cluster heatmap. (**C**) Protein expression difference cluster heatmap. (**D**) Metabolic pathway difference cluster heatmap. Soil rhizosphere microbial functions, encompassing KEGG predictions, enzyme activities, protein expression, and metabolic pathways, are downregulated with the reduction in rhizosphere metabolites. CKG (rhizosphere of control ), SWTG (rhizosphere of biochar), CKF (non-rhizosphere of control), and SWTF (non-rhizosphere of biochar).

We further investigated functional variations in the soil microbial community by tagging gene sequences to predict practical abundance using PICRUSt functional prediction, which has a high prediction accuracy that can reach more than 85–90 percent for soil flora. The results reveal that soil microbial community functions tended to decrease with the biochar strategy, and most were downregulated, according to KEGG prediction, protein function, enzyme activity, and metabolic pathway analyses (Figure 4 and Figure S2).

Metabolic functions were downregulated under the biochar strategy (SWTG) (Figure 3 and Figure S2). These functions encompassed various processes, such as signaling molecules and interactions, the metabolism of terpenoids and polyketides, transport and catabolism, amino acid metabolism, lipid metabolism, biosynthesis of other secondary metabolites, nucleotide metabolism, and energy metabolism (Figure 4A). Furthermore, cellular processes and genetic information showed signs of downregulation, particularly in processing signaling molecules and interactions, signal transduction, and membrane transport. However, the cellular community of prokaryotes was downregulated, while that of eukaryotes was upregulated (Figure 4A).

However, we detected upregulation during our investigation, specifically in the form of stimulated enzyme expression and activity, e.g., formate dehydrogenase (NADP(+)) and ATP citrate synthase (NADP(+)) (Figure 3B). The expression of proteins, e.g., the hydrogenase-4 membrane subunit HyfE, the SHS2 domain protein implicated in nucleic acid metabolism, formate hydrogenlyase subunit 4, the coenzyme F420-reducing hydrogenase-gamma subunit, glutamate formiminotransferase, and the Perexiredoxin family protein, was upregulated (Figure 4C). Metabolic pathways that were upregulated included sucrose biosynthesis I (from photosynthesis), L-isoleucine brothers IV, the incomplete reductive TCA cycle, and the reductive acetyl coenzyme A pathway (Figure 4D). Overall, reduced rhizosphere metabolites downregulated most microbial community functions in the rhizosphere.

### 3.4. Phenolic Acids Decrease in the Rhizosphere

We initially speculated that biochar adsorbs root secretions, as previously reported. To validate our hypothesis, we performed targeted metabolomics on the biochar interlayer (biochar-only) strategy and determined its ability to absorb metabolites and which metabolites were most affected.

**Figure 5:**
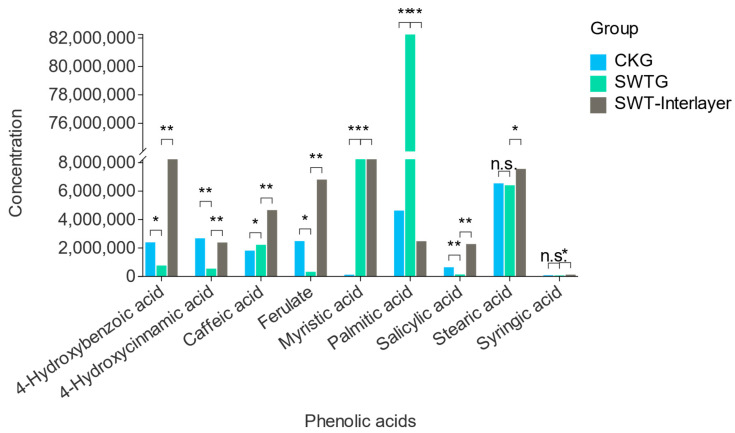
Targeted phenolic acid concentration comparison. *, *p* < 0.05; **, *p* < 0.01; ***, *p* < 0.001.

We found that most metabolites were identified and targeted within the biochar strategy interlayer. In support of our hypothesis, the biochar strategy had fewer metabolites in the rhizosphere but more metabolites in the interlayer of the plants in the biochar strategy (Figure 5). Notably, the metabolites that were adsorbed were phenolic acids with autotoxic effects, e.g., 4-hydroxybenzoic acid, 4-hydroxycinnamic acid, ferulate, stearic acid, and salicylic acid, indicating that biochar can also reduce root autotoxins (Figure 5).

Consequently, as previously reported, our original prediction was that the experiment would definitely reduce rhizosphere metabolites, accompanied by a decrease in root autotoxins in the rhizosphere soil. As a result, this reduction in autotoxins optimizes the rhizosphere microbial ecology by creating a healthier environment for microorganisms to thrive.

### 3.5. Fungal Biomarkers Play Competitive Roles in Microbial Interactions

Furthermore, we detected positive correlations between fungi in the biochar-induced rhizosphere, i.e., between *Monographella*, *Acremonium*, *Geosmithia*, and *Funneliformis*. However, they were negatively correlated with 60 percent of the Top 20 differential fungi and were not significantly negatively correlated with fungal biomarkers in the control of rhizosphere.

**Figure 6:**
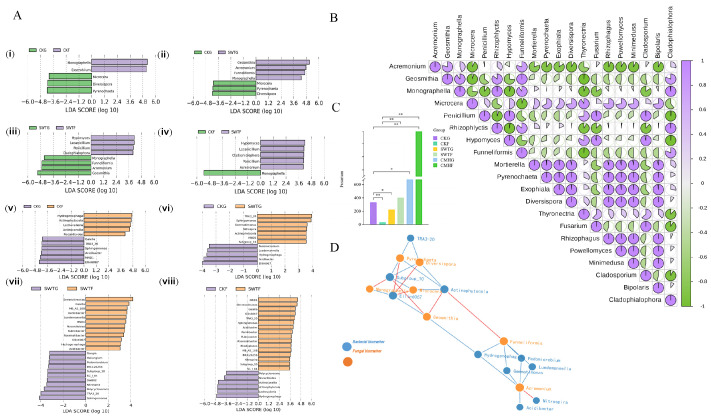
LEfSe analysis and correlation of biomarkers in soil microbiomes. (**A**) (**i**–**viii**). Discriminating fungal and bacterial microorganisms according to LEfSe analysis. In the biochar strategy, the fungal biomarkers *Monographella*, *Acremonium*, *Geosmithia*, and *Funneliformis* were significantly enriched. (**B**) Correlation analysis of the Top 20 most abundant fungi. *Acremonium*, *Geosmithia*, and *Funneliformis* exhibit negative correlations with more than 60 percent of the Top 20 most abundant fungi. (**C**) Histogram of the pathogen abundance of *Fusarium* sp. A marked decrease in *Fusarium* sp. is observed in the biochar-amended rhizosphere (SWTG). (**D**) The interomics correlation network of fungal and bacterial biomarkers. The fungal biomarkers are positively intercorrelated and negatively correlated with bacterial biomarkers. *, *p* < 0.05; **, *p* < 0.01.

In addition, the biochar strategy (SWTG) had more bacterial biomarkers than the control (Figure 6A(vii,viii)), which could attract different species of functional bacteria for colonization (Figure 6A(vi,viii)). In addition to the *Proteobacteria* and *Actinobacteria* phyla, the bacterial biomarkers collected in this study (Figure 6A(vii)) included the *Gemmatimonadota* and *Nitrospirota* phyla, which have a wide range of biomarker sources (Figure 6A(vii)).

To gain a better understanding of the interactions between biomarkers among community members, an interomics correlation network was constructed between biomarkers found in fungi and bacteria. We found strong negative correlations between fungal biomarkers, i.e., *Acremonium* and *Funneliformis*, and bacterial biomarkers, particularly those found in the control rhizosphere, even stronger than the positive correlations with fungal and bacterial biomarkers within the same biochar-amended rhizosphere.

Taken together, our findings imply that critical fungal biomarkers with reduced rhizosphere metabolites potentially play a competitive role in microbial interactions within the soil rhizosphere.

We conducted a study on the pathogenic fungal *Fusarium* sp. Although not identified as a biomarker, controlling its growth is crucial for restoring the balance of rhizosphere microbial ecology. Our results show that the biochar strategy resulted in a statistically significant decrease in the abundance of *Fusarium* sp. (*p* < 0.05) (Figure 6C); conversely, the grass ash strategy resulted in a significant increase (Figure 6C), which may explain the phenotypic superiority of the biochar strategy to grass ash.

### 3.6. Ecological Roles of Biomarker Microorganisms in the Rhizosphere

To better understand the role of biomarker microorganisms in optimizing rhizosphere microbial ecology, we also propose a framework for how biomarker microorganisms play roles in three major segments, i.e., microbial networks, metabolites, and soil nutrients.

**Figure 7:**
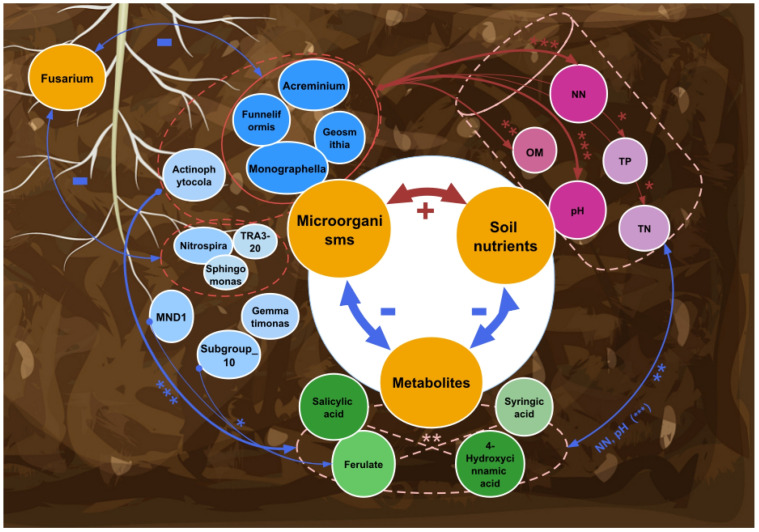
Network interaction maps between microorganisms, metabolites, and soil nutrients. Biomarker microorganisms were positively correlated with soil nutrients and elevated pH but negatively correlated with phenolic acid (*p* < 0.001). Both fungal and bacterial biomarker microorganisms were negatively correlated with the pathogen *Fusarium*. NN, nitrate nitrogen; OM, organic matter; TP, total phosphorus; TN, total nitrogen. *, *p* < 0.05; **, *p* < 0.01; ***, *p* < 0.001; Blue arrows: negative correlation; red arrows: positive correlation; line thickness indicates correlation strength.

We found that fungal biomarkers were positively correlated with soil nutrients and elevated pH but negatively correlated with the pathogenic fungus *Fusarium* sp. and phenolic acids, which can be autotoxic to the root system. Therefore, the ecological functions of the fungal biomarkers were more significant in increasing nutrients and decreasing autotoxicity and soil acidification (Figure 7).

Bacteria from the genus *Acreminium* work synergistically with fungi to perform similar functions. Furthermore, nitrate nitrogen and pH were the most critical factors determining the rhizosphere soil environment (Figure 7).

Overall, biomarker microorganisms play a significant ecological role in optimizing the rhizosphere environment by efficiently utilizing soil nutrients, competing with pathogens, and participating in the metabolic function of reducing autotoxic metabolites.

## 4. Discussion

The biochar-induced rhizosphere shift in fungal diversity from greater to less could redefine plant–microbe symbiosis in the rhizosphere. Typically, the rhizosphere is a hotspot for microbial diversity due to the attractant properties of root exudates [19]. However, our studies show that biochar application triggers a counterintuitive effect: a decrease in fungal diversity in the rhizosphere and a concurrent increase in the surrounding soil. This biochar-induced diversity shift reduces fungal colonization and pathogen invasion, addressing a prevalent challenge in soils prone to continuous cropping obstructions (CCOs), where pathogenic fungi often prevail [20]. Our findings point to a novel strategy for enhancing soil resistance to CCOs, thereby benefiting sustainable agriculture by disrupting pathogenic dominance and rebalancing the soil microbiome to support plant health and disease resistance.

Notably, the biochar-induced rhizosphere would inhibit the synergistic damage of root secretions with pathogens. Our research indicates that biochar not only reduces fungal diversity but also restructures the rhizosphere community, notably by suppressing pathogenic *Fusarium* sp. The secretion of phenolic acids, while a plant defense mechanism, can inadvertently nourish soil-borne fungi, leading to a cycle of acidification and immune suppression [21]. Our findings suggest that reducing phenolic acids can curb pathogen virulence by depriving them of vital nutrients. This targeted approach to soil management disrupts the environment that supports pathogens, offering a new strategy for soil health and agricultural resilience against CCOs. By modulating the composition of root exudates, we can tip the balance in the rhizosphere in favor of beneficial microbes over pathogens, laying the groundwork for innovative soil management practices.

However, the explicit consideration of fundamental ecological processes for developing complex microbial communities is in its infancy. Our work reveals that higher bacterial diversity is indicative of a robust ecosystem that can outcompete pathogens for resources, thus preserving ecosystem health [22]. Soils with greater microbial diversity tend to have more ecological functions and greater resistance to environmental stress. Communities with high diversity have high resource utilization complementarity to use resources in the environment entirely, which leaves fewer resources available for pathogens to invade [23], ultimately helping the ecosystem to remain healthy.

Within the core microbiota, “biomarker microorganisms” can influence the community structure through biotic solid interactions with the host or with other microbial species [24], and these interactions function as the first line of defense against pathogens, as their removal results in the loss of interactions—for example, with Enterobacter cloacae [25]. Identifying “key microorganisms” within this community is essential for understanding their role in enhancing the rhizosphere. We have identified key biomarkers—*Monographella*, *Acremonium*, *Geosmithia*, and *Funneliformis*—that correlate negatively with other microbes, suggesting their role in competitive interactions. These markers optimize microbial ecology by mobilizing soil nutrients, curbing pathogen growth, and reducing phenolic acids. Our results suggest a framework for synthetic microbial communities to fine-tune ecological functions in the rhizosphere.

Our research results show that the rhizosphere shift recruited *Funneliformis* spp., a type of arbuscular mycorrhizal fungi (AMF), enriched the soil environment, and enhanced plant growth by increasing nutrient uptake, particularly phosphorus, and improving soil microbial diversity [26]. This symbiotic relationship boosts plant biomass and health while also mitigating the challenges of continuous cropping, such as soil degradation and disease. By promoting soil enzyme activity and plant resistance to environmental stresses, including heavy metals, *Funneliformis* spp. play a crucial role in fostering soil fertility and supporting sustainable agricultural practices [27].

This research enhances our understanding of leveraging microbial interactions to improve the health and productivity of agro-ecosystems [28]. The development and application of synthetic microbial communities (SynComs) in plants provide a novel avenue for understanding the complex dynamics of plant microbiome interactions, including community assembly and member relationships [29]. Integrating omics-based predictions with SynCom-based microbial interactions illuminates how microbial consortia can be manipulated to combat soil-borne diseases and optimize rhizosphere microbial ecology globally, paving the way for innovative soil management strategies powered by the plant microbiome [30].

In conclusion, a sophisticated understanding of rhizosphere metabolites and their role in shaping microbial communities is pivotal for advancing sustainable agricultural practices and promoting soil and plant health. This knowledge is essential for developing innovative strategies that harness the power of the plant microbiome to enhance soil health and agricultural sustainability.

## Data Availability

The original contributions presented in the study are included in the article/supplementary material, further inquiries can be directed to the corresponding authors.

## Supplementary Materials

The following supporting information can be downloaded at https://www.mdpi.com/article/10.3390/microorganisms12122420/s1, Figure S1: Plant growth and seedling survival in different strategies; Figure S2: KEGG prediction difference clustered heatmap; Figure S3: Comparative analysis of physicochemical properties of rhizosphere soil and correlation analysis between differential microbes and soil physicochemical properties.
