# Supplementary material for: Rhizosphere Shifts: Reduced Fungal Diversity and Microbial Community Functionality Enhance Plant Adaptation in Continuous Cropping Systems
Source: Microorganisms. 2024 Nov 25;12(12):2420. doi: 10.3390/microorganisms12122420 (PMC11678050; doi:10.3390/microorganisms12122420)
Supplement: Supplementary file 1 [file microorganisms-12-02420-s001.zip › microorganisms-3260351-supplementary.pdf]

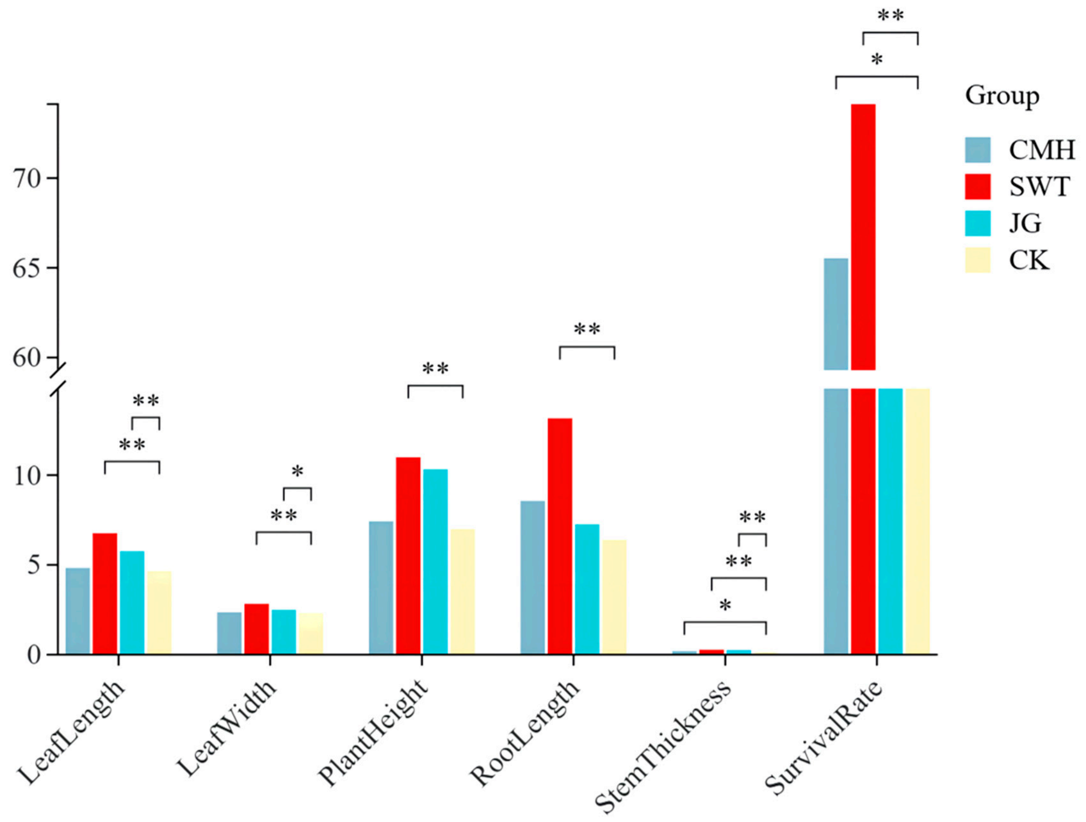

Supplementary Figure S1. Plant growth and seedling survival in different strategies. CMH (grass ash), SWT (biochar), JG (corn stover), and CK (control).Plant growth and seedling survival were highest in the biochar strategy(P<0.01). "*, P < 0.05; **, P < 0.01".

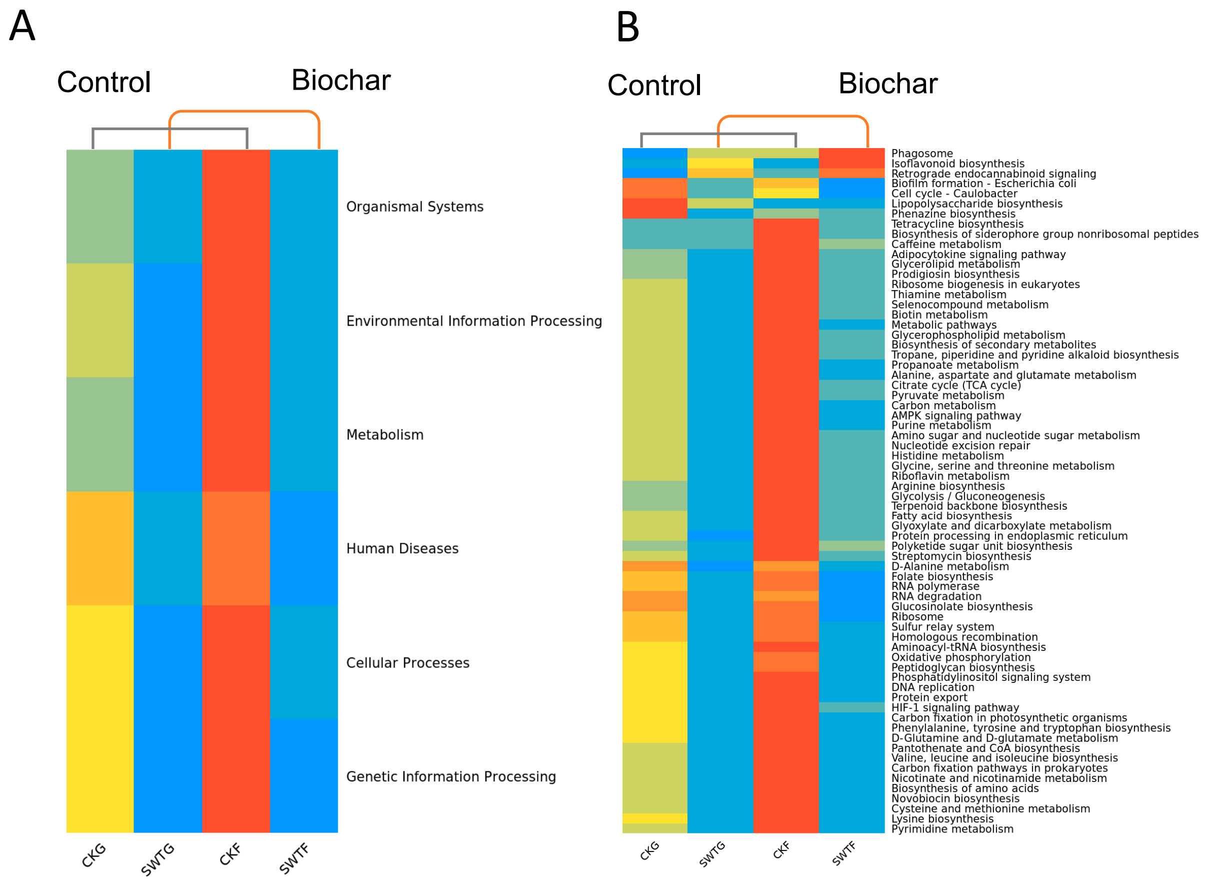

Supplementary Figure S2. KEGG prediction difference clustered heatmap (A level1, B level3).

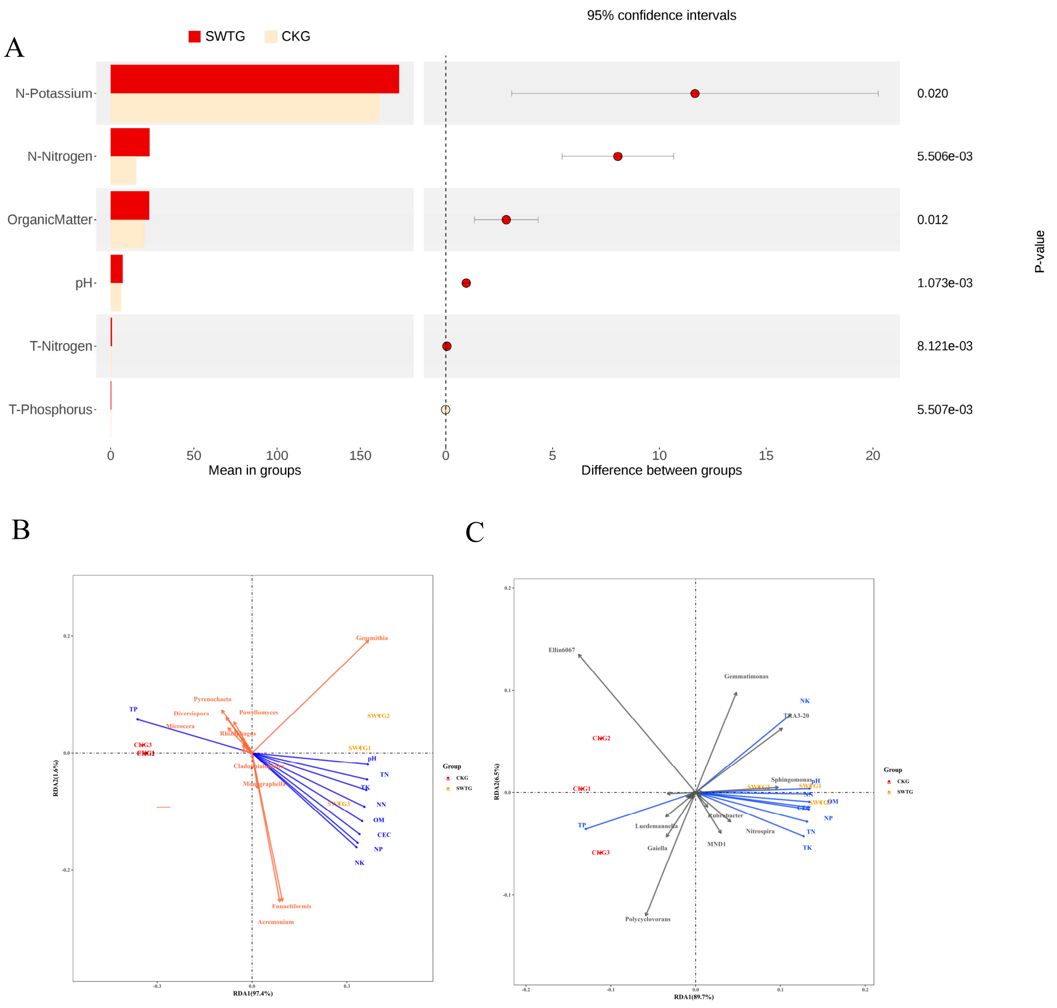

Supplementary Figure S3. Comparative analysis of physicochemical properties of rhizosphere soil and correlation analysis between differential microbes and soil physicochemical properties. (**A**) Comparative analysis of physicochemical properties of rhizosphere soil after biochar addition and continuous cropping soil planting. (**B**) Correlation analysis between soil physicochemical properties and differential fungi of after biochar addition and continuous cropping soil planting. (**C**) Correlation analysis between soil physicochemical properties and differential bacteria of after biochar addition and continuous cropping soil planting.
